# Learned predators enhance biological control via organizational upward and trophic top‐down cascades

**DOI:** 10.1111/1365-2664.13791

**Published:** 2020-11-16

**Authors:** Peter Schausberger, Demet Çekin, Alena Litin

**Affiliations:** ^1^ Department of Behavioral and Cognitive Biology University of Vienna Vienna Austria; ^2^ Group of Arthropod Ecology and Behavior Department of Crop Sciences University of Natural Resources and Life Sciences Vienna Austria

**Keywords:** learning, pest management, predatory mites, thrips, trophic cascades

## Abstract

1. Learning is a behavioural change based on memory of previous experiences and a ubiquitous phenomenon in animals. Learning effects are commonly life‐stage‐ and age‐specific. In many animals, early life experiences lead to pervasive and persistent behavioural changes.
2. There is broad consensus that learning has far‐reaching implications to biological control. Proximate and ultimate factors of individual learning by parasitoids and true predators are relatively well understood, yet the consequences of learning to higher organizational levels, populations and communities, and top‐down trophic cascades are unexplored.
3. We addressed this issue using a tri‐trophic system consisting of predatory mites *Amblyseius swirskii*, Western flower thrips *Frankliniella occidentalis* and whole common bean plants, *Phaseolus vulgaris*. *F. occidentalis* are notorious horticultural pests that are difficult to control. Therefore, practitioners have much to gain by optimizing biological control of thrips.
4. Previous studies have shown that early life experience of thrips by *A. swirskii* improves foraging on thrips later in life due to decreased prey recognition times and increased predation rates, together enhancing predator fecundity. Here, we hypothesized that early learning by *A. swirskii* enhances biological control of thrips via immediate and cascading effects. We predicted that release of thrips‐experienced predators enhances predator population growth and thrips suppression and reduces plant damage as compared to release of thrips‐naïve predators.
5. The behavioural changes brought about by early learning cascaded up to the population and community levels. Thrips‐experienced predators caused favourable immediate and cascading effects that could not be compensated for in populations founded by thrips‐naïve predators. Populations founded by thrips‐experienced predators grew faster, reached higher abundances, were more efficacious in suppressing an emerging thrips population and kept plant damage at lower levels than populations founded by thrips‐naïve predators. Plant fecundity correlated negatively with thrips abundance and positively with predatory mite abundance. Improved biological control was mainly due to thrips‐experienced founders providing for a head‐start in predator population growth and thrips suppression.
6. *Synthesis and applications*. Our study suggests that learned natural enemies have high potential to optimize augmentative biological control on a larger scale due to favourably modulating organizational upward and trophic top‐down cascades.

## Graphical abstract

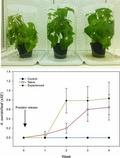

## 1 INTRODUCTION

Learning, that is, experience‐based change in behaviour, is a ubiquitous phenomenon in animals and relevant in all major behavioural contexts such as foraging, reproduction and social interactions (Alloway, 1972; Byrne, 2017; Papaj & Lewis, 1993; Pearce, 2013; Smid & Vet, 1986). Learning allows individuals to flexibly respond to environmental variation and adaptively adjust their behaviours (Dukas, 2008). The effect size of experience and learning intensity are commonly age‐, life‐stage‐ and phase‐specific (Little et al., 2019; Stamps & Krishnan, 2017). In many animals, it is especially experiences made in the early phases of life, when the neural system is more plastic than later on, that produce profound and persistent changes in behavioural trajectories (Rohlfs Dominguez, 2014; Schausberger et al., 2018; Stamps, 2016). Learning is an individual‐level trait yet the effects of individual‐based learning may have important consequences at higher organizational levels such as populations and communities (e.g. Ishii & Shimada, 2012; Lister, 2014; Willett et al., 2017) and may decisively shape top‐down trophic cascades (Hairston et al., 1960).

Learning may alter numerous aspects of foraging behaviour and thus has obvious implications to the use of natural enemies in biological control (for review, see Little et al., 2019; Prokopy & Lewis, 1993). Regarding biological control, we have a relatively good understanding of learning by predators and parasitoids at the individual level such as the types of learning that are at play, the cues that are learned, and life stage and age dependency of learning (for review, see Giunti et al., 2015; Little et al., 2019). However, while individual‐based learning has been well researched for various parasitoid and predator species in diverse behavioural contexts (for review, see Giunti et al., 2015; Kruidhof et al., 2019; Little et al., 2019; Papaj & Lewis, 1993), evidence of organizational scale cascades, as shaped by the release of learned natural enemies, is lacking. Similarly, top‐down trophic cascades of learned natural enemies on host plant damage and fitness remain elusive. Lack of research scrutinizing organizational up‐scaling and trophic down‐cascading of learning is possibly due to the widespread assumption that experience‐based behavioural changes attenuate with accumulating experiences over time and cancel out in the next generation, because of the released individuals' offspring making their own experiences. However, early life experiences may lead to pervasive and persistent, sometimes lifetime, changes that cannot be compensated for by experiences made later in life (Immelmann, 1975; Monaghan, 2008; Schausberger et al., 2010, 2018; Schausberger & Peneder, 2017; Stamps & Krishnan, 2017). Importantly, individual learning may decisively influence population and community dynamics if, for example, released learned individuals have an improved prey/host finding and predation/parasitization capacity and especially if such improved predation/parasitization performance translates into enhanced reproduction (Christiansen et al., 2016). Albeit not looking into longer‐term dynamics, a classic population‐level example comes from Hare et al. (1997), who showed that groups of target host species‐conditioned parasitoids *Aphytis melinus,* released against California red scale *Aonidiella aurantii* on lemon trees, left more offspring than groups of target host‐naïve parasitoids. Along the same line, Masry et al. (2019) recently showed that parasitoids that were conditioned to different fruit odours in the laboratory, and were then released in groups in the field, visited more likely fruits matching their previous experience, and thus left more progeny there, than they visited unfamiliar fruits. In addition to immediately apparent community effects such as stronger prey/host suppression and higher natural enemy progeny production, learned natural enemies may also influence community dynamics via subtle trans‐generational effects (Bonduriansky & Day, 2009; Mousseau & Fox, 1998). Parental experiences may influence the physiological state and behavioural and ecological performance of offspring, that is, how offspring interact with their environment and process environmental stimuli, including the way of processing own prey experiences (Reichert et al., 2017; Seiter & Schausberger, 2015).

Here, we addressed organizational up‐scaling and trophic top‐down cascading of early learning by natural enemies. Using a tri‐trophic system consisting of predatory mites *Amblyseius swirskii*, herbivorous Western flower thrips *Frankliniella occidentalis* and common bean plants *Phaseolus vulgaris,* we assessed whether and how individual predator‐based early learning cascades up to the population and community levels and exerts top‐down trophic effects on plant damage and fecundity. This tri‐trophic system prevails in natural as well as in agricultural and horticultural settings. *Amblyseius swirskii* are natural enemies of various herbivorous mite and insect pests such as spider mites, whiteflies and thrips, and are accordingly exploited in biological control in diverse agricultural and horticultural crops (Arthurs et al., 2009; McMurtry & Croft, 1997; Messelink et al., 2008; Nomikou et al., 2001). *Amblyseius swirskii* can also feed on plant‐derived substances such as pollen (Goleva & Zebitz, 2013; Nguyen et al., 2013). Due to its generalized feeding habits and widespread use in biological control, *A. swirskii* is an especially suitable predator for the scope of our study. Thrips are still the most difficult to control herbivorous pests of horticultural crops despite longstanding and ever‐increasing efforts to identify novel natural enemy species and/or to implement novel control strategies (Mouden et al., 2017). Difficulties in thrips control are due to a number of biological and ecological factors such as high adaptability and high phenotypic plasticity, their life cycle partly occurring on green plant parts and in the soil, their opportunistic life style allowing them to feed on both plant and animal food and their well‐developed abilities to defend themselves against natural enemies (Morse & Hoddle, 2006; Reitz, 2009).

Similar to other predatory mite species such as *Phytoseiulus persimilis* and *Neoseiulus californicus* (Rahmani et al., 2009; Schausberger & Peneder, 2017; Seiter & Schausberger, 2015), *A. swirskii* possesses well‐developed learning abilities. Both non‐associative (unrewarded; only exposed to prey or their cues but no feeding on prey) and associative (rewarded; exposed to prey and feeding on prey) prey experiences during early life of the predators result in profound and persistent enhancement of predation of familiar prey later in life. Enhanced predation is evident in shortened attack latencies, which increase prey profitability (Stephens & Dunlap, 2017), and higher predation rates, which, in turn, increase the reproductive output (Christiansen & Schausberger, 2017; Christiansen et al., 2016; Reichert et al., 2017; Schausberger et al., 2018, 2020; Seiter & Schausberger, 2016). We hypothesized that the behavioural changes brought about by the predators' early life experience of thrips as prey cascade up to the population and community levels and enhance the predators' efficacy in biological control of thrips, relative to thrips‐naïve predators. The plant may benefit from learned predators via a top‐down trophic cascade. We predicted that (a) thrips‐experienced predators enhance biological control of thrips via immediate and cascading effects, (b) release of thrips‐experienced predators allows faster population growth and higher population levels of the predators than release of thrips‐naïve predators, (c) increased predator abundance results in decreased thrips abundance and (d), (a), (b) and (c) together translate into decreased plant damage and increased plant fitness.

## 2 MATERIALS AND METHODS

### 2.1 Experimental animals, origin and rearing

Predatory mites *Amblyseius swirskii* used in experiments were derived from a population reared in the laboratory on two‐spotted spider mites *Tetranychus urticae*. The predatory mite population had been founded by around 200 specimens collected on citrus trees in Israel about 3 years before starting the experiments. In the laboratory, the predators were continuously maintained on artificial arenas, consisting of acrylic tiles (14 × 14 cm) resting on water‐saturated foam cubes inside plastic trays (20 × 20 cm) half‐filled with water, and fed mixed stages of *T. urticae*. The spider mites were reared on whole common bean plants, *Phaseolus vulgaris*, in an air‐conditioned greenhouse cabinet and brushed from infested leaves onto arenas in 3‐ to 4‐day intervals.

Western flower thrips *Frankliniella occidentalis* was continuously reared in the laboratory on whole green bean pods inside glass jars closed by gauze. The laboratory population had been founded by around 100 specimens collected from chrysanthemum flowers at Keele University, UK, half a year before starting the experiment. Adult thrips females used to infest the experimental plants were directly withdrawn from the rearing. To obtain first and second stage larvae of thrips, used for conditioning the predatory mites, adult thrips females were randomly withdrawn from the rearing and placed onto detached primary bean leaves (~11 × 13 mm) resting adaxial side down on a 5% agar solution in a closed petri dish (14 cm Ø, 2 cm height). For ventilation, the lid of the petri dish had a circular opening (1 cm Ø) covered with gauze (mesh openings 50 µm). Thrips females were allowed to oviposit on the bean leaf for 24 hr. After removing the thrips females, the petri dish was stored in a climate chamber at 25 ± 1°C, 65 ± 5% relative humidity (RH) and 16:8 hr L:D photoperiod for 4–5 days. At that time, most larvae had hatched and the petri dish was kept in a fridge at 8°C and constant darkness to stop any further development of the larvae.

### 2.2 Pre‐experimental procedures

In the experiment, we used thrips‐naïve and thrips‐experienced predatory mites. To generate thrips‐naïve and ‐experienced predators, the predators were housed inside acrylic cages from the egg until the early protonymphal stage. Each acrylic cage consisted of a cylindrical cavity (1.5 cm Ø, 0.3 cm height) drilled into a rectangular acrylic plate closed at the bottom by gauze (mesh openings 50 µm) and on the upper side by a microscope slide. Inside cages, thrips‐naïve predators were provided with pollen of cattail *Typha angustifolia* (Nutrimite; Biobest, Belgium), whereas thrips‐experienced predators were provided with first and second stage larvae of thrips, which had been killed by deep‐freezing. Inside cages, both thrips‐naïve and thrips‐experienced predators had also access to free water via water‐saturated filter paper strips (Schausberger, 1997). Late protonymphs of thrips‐naïve and ‐experienced predators were transferred from the cages onto separate detached bean leaf arenas dusted with cattail pollen and reared there until they had reached adulthood and were mated. Cages and detached leaves were stored in a climate chamber at 23 ± 1°C, 40%–60% RH and 16:8 hr L:D. Gravid females and adult males that had experienced either pollen (for thrips‐naïve predators) or thrips (for thrips‐experienced predators) in early life were used in the experiment.

### 2.3 Experimental procedure

The biological control efficacy of thrips‐naïve and ‐experienced *A. swirskii* was compared on whole thrips‐infested common bean plants. Three bean plants (*P. vulgaris* var. Maxi), which were grown in a nutrient‐rich peat moss sand substrate mixture in a 14 cm Ø pot, represented one experimental plant unit (replicate). Each plant unit was placed in a plastic tray (styrene‐acrylonitrile; 80 × 30 × 2.5 cm) and covered by an acrylic cylinder (40 cm high, 25 cm Ø, 0.4 cm wall thickness). The top of the cylinder was closed by a thrips‐ and mite‐proof gauze (mesh openings 50 µm). The bottom rim of the cylinder was lubricated with vaseline to provide a tight attachment to the tray. The cylinder prevented any immigration and emigration of thrips and mites from/into experimental units. Plants did not receive any fertilizer and were watered as needed during the experiment. Experimental units were placed inside a climate chamber with environmental conditions set at 23 ± 1°C, 40%–60% relative humidity and 16:8 hr L:D.

The experiment was started when the primary leaves and first trifoliate leaves of the experimental bean plants were fully developed (approximately 3–4 weeks after seeding). To start the experiment (week 2, i.e. two weeks before leaf sampling started in week 0), the plants were slightly dusted with cattail pollen by thrice dipping a marten's hair brush (size 0) into pollen and evenly sprinkling the pollen onto the leaves of the experimental bean plants. Pollen was provided to enhance *A. swirskii's* establishment on plants (Nguyen et al., 2013; Pijnakker et al., 2016). To infest the plants with thrips, three adult thrips females were randomly withdrawn from the rearing, placed inside cylindrical glass tubes (2.7 cm Ø, 6 cm high) and the glass tubes placed beneath the acrylic hoods covering the plants. By doing so, the thrips females could naturally disperse from the glass tubes to the plants. Two weeks later (week 0), the experimental units were randomly assigned to three treatments concerning predatory mite release: no predatory mites (control), thrips‐naïve predatory mites and thrips‐experienced predatory mites. Predatory mite releases consisted of three gravid females and one adult male (~3–5 days after moulting to adult and mating) per experimental unit, which were either thrips‐naïve or thrips‐experienced. Mites were picked from leaf arenas and released on the surface of leaves using a moistened marten's hair brush. Control treatments received sham releases, that is, acrylic hoods were opened and plants handled in the same way as the units receiving mites except that leaves were touched with a moistened brush devoid of mites. Each treatment was replicated 15 times (15 experimental units each consisting of a pot harbouring three bean plants per treatment). Sampling started in week 0 (just before the predatory mite release) and continued until week 4. Each week, one fully developed trifoliate leaflet was sampled (clipped off plants) from each experimental unit. Each leaf was checked under a stereo‐microscope to record the number and life stages of thrips and predatory mites and to determine the percentage of leaf area damaged by thrips. To count the number of thrips eggs, which are inserted in the leaf tissue, the leaves were soaked in denatured absolute ethanol until chlorophyll was washed out. Thrips eggs are easily visible inside chlorophyll‐drained leaves. Care was taken to clip off a leaflet from a different trifoliate within each experimental unit each week. In week 4, all green bean pods were harvested and counted.

### 2.4 Statistical analyses

IBM SPSS Statistics 26 (IBM) was used for statistical analysis. Mean and time‐dependent abundance of thrips and predatory mites and proportional leaf damage caused by thrips in weeks 1–4 (time specified as nested auto‐correlated within‐subject factor) were compared among treatments by separate generalized estimating equations (GEE; Gamma distribution *x* + 1, identity link for thrips and mites; normal distribution with identity link for leaf damage). Proportional leaf damage (i.e. the proportion of leaf damaged by thrips) was arcsine square‐root transformed before analysis. Similarly, mean and time‐dependent life‐stage‐specific abundance of thrips (eggs, larvae and adults) was compared among treatments by GEE (Gamma distribution *x* + 1, identity link). In case GEE revealed significant treatment effects, least significant difference (LSD) tests were used to separate the means. To assess the effects of thrips and predatory mites on plant fitness (fecundity), we analysed the relation between the number of green pods produced by plants and the number of thrips (+1) and predatory mites (+1) observed on plants in weeks 3 and 4 by logarithmic regression.

## 3 RESULTS

**Figure 1:**
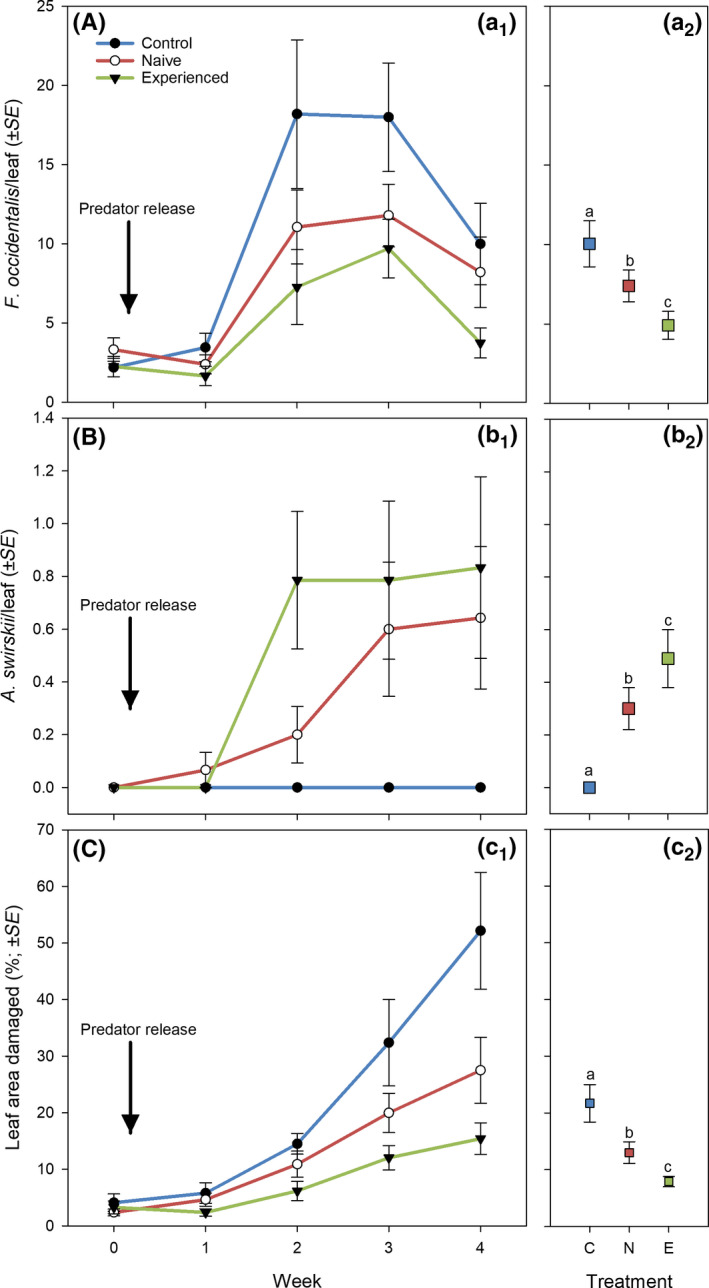
Time‐specific (a_1_, b_1_c_1_) and pooled mean (a_2_, b_2_, c_2_) abundance of thrips*Frankliniella occidentalis*(A) and predatory mites*Amblyseius swirskii*(B) and percentage leaf damage caused by thrips (C) on bean plants. Bean plants were artificially infested by thrips in week 2 and thrips‐naïveand ‐experiencedpredatory mites were released in week 0. The control treatmentdid not receive any predatory mites

**Figure 2:**
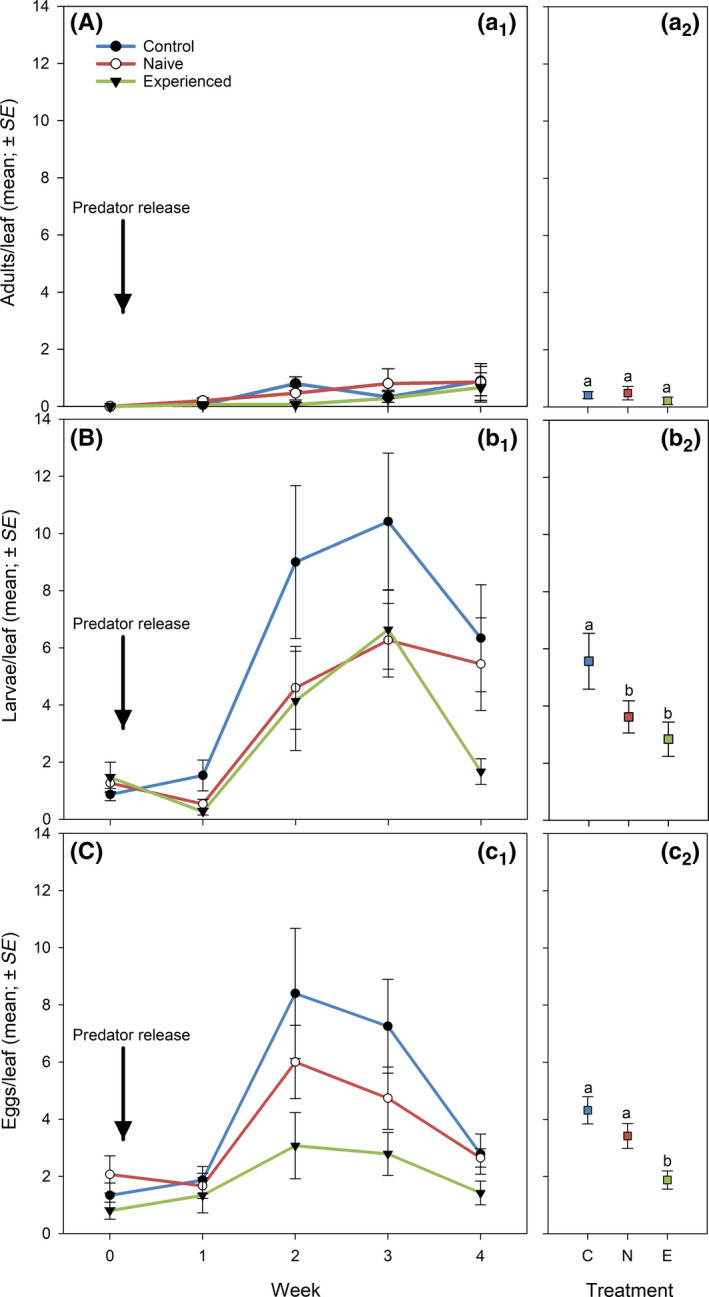
Time‐specific (a_1_, b_1_, c_1_) and pooled mean (a_2_, b_2_, c_2_) life‐stage‐specific abundance of adults (A), larvae (B) and eggs (C) of thrips*Frankliniella occidentalis*on bean plants. Bean plants were artificially infested by thrips in week 2 and thrips‐naïveand ‐experiencedpredatory mites were released in week 0. The control treatmentdid not receive any predatory mites

**Figure 3:**
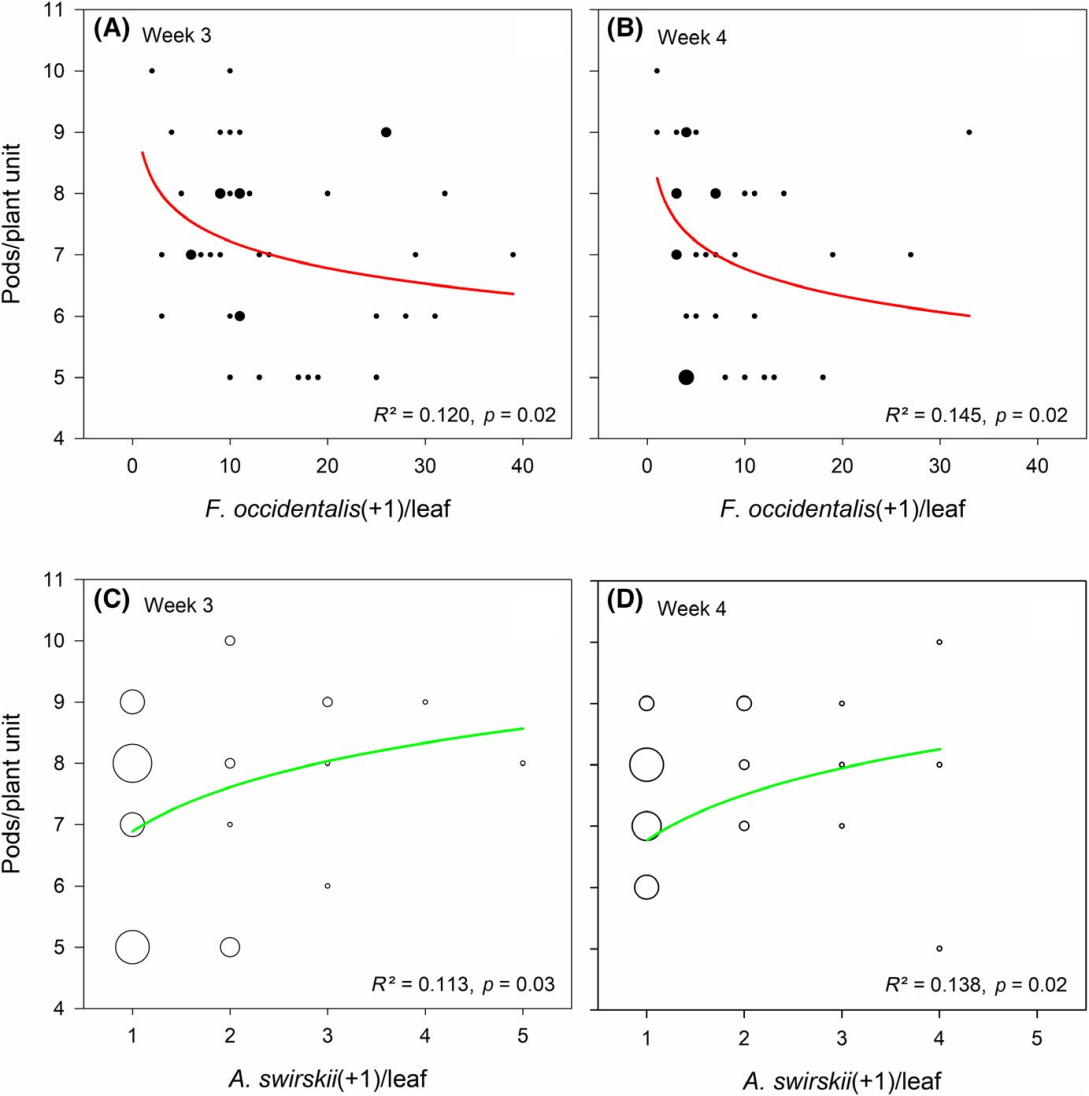
Logarithmic regression of the number of pods produced by bean plants on the number of thrips*Frankliniella occidentalis*(A, B) and predatory mites*Amblyseius swirskii*(C, D) in weeks 3 and 4 (all treatments pooled). Bubble size scaled with sample size

Both grand mean (Wald $\chi_{2}^{2}$ = 19.872, *p* < 0.001) and time‐dependent (Wald $\chi_{9}^{2}$ = 86.464, *p* < 0.001) abundance of thrips and grand mean (Wald $\chi_{2}^{2}$ = 32.281, *p* < 0.001) and time‐dependent (Wald $\chi_{9}^{2}$ = 35.613, *p* < 0.001) abundance of predatory mites differed significantly among treatments (Figure 1A,B). Also, both grand mean (Wald $\chi_{2}^{2}$ = 32.435, *p* < 0.001) and time‐dependent (Wald $\chi_{9}^{2}$ = 113.701, *p* < 0.001) leaf damage caused by thrips differed significantly among treatments (Figure 1C). Thrips had the lowest abundance, predatory mites had the highest abundance and leaf damage was the least, in the thrips‐experienced predator treatment, followed by the thrips‐naïve predator treatment, followed by the control treatment without predators (LSD; *p* < 0.05 for every pairwise comparison; Figure 1A–C). Scrutiny of the time‐dependent parameter estimates within treatments revealed that thrips abundance remained at the same level in weeks 3 and 4 in the control and thrips‐naïve predator treatments (*p* > 0.05), whereas it decreased significantly from week 3 to 4 in the thrips‐experienced predator treatment (*p* < 0.05). Regarding life‐stage‐specific thrips abundance, the differences among treatments were most pronounced in the egg stage (grand mean: Wald $\chi_{2}^{2}$ = 13.642, *p* = 0.001; time‐dependent: Wald $\chi_{9}^{2}$ = 94.820, *p* < 0.001) followed by larvae (grand mean: Wald $\chi_{2}^{2}$ = 10.875, *p* = 0.008; time‐dependent: Wald $\chi_{9}^{2}$ = 263.101, *p* < 0.001; Figure 2A–C). The grand mean of adult thrips did not differ among treatments (Wald $\chi_{2}^{2}$ = 2.065, *p* = 0.35) but the numbers developed differently over time (Wald $\chi_{9}^{2}$ = 47.059, *p* < 0.001; Figure 2A). Larvae abundance was similar in the thrips‐naïve and ‐experienced predator treatments (*p* = 0.12) and significantly lower in the predator treatments than the control treatment without predators (*p* < 0.05 for every pairwise comparison; Figure 2B). Egg abundance was lower in the thrips‐experienced predator treatment than in the thrips‐naïve predator and control treatments (LSD; *p* < 0.02 for every pairwise comparison), which did not differ from each other (*p* = 0.19; Figure 2C). Logarithmic regressions revealed significant correlations between the number of bean pods produced and the number of thrips (negative; Figure 3A,B) and the number of predatory mites (positive; Figure 3C,D) in weeks 3 and 4.

## 4 DISCUSSION

Our study documents how early life experiences by natural enemies can modulate organizational upward cascades and trophic top‐down cascades in a tri‐trophic system. Learned predatory mites induced faster population growth and allowed higher population levels, suppressed herbivorous thrips populations more strongly and kept plant damage caused by thrips at lower levels than did thrips‐naïve predatory mites. The abundance of predatory mites correlated positively, whereas thrips abundance correlated negatively, with the number of fruits produced by the host plants.

Our study provides proof of principle for favourable effects of learning by natural enemies in augmentative biological control. While the significance of learning by pests and their natural enemies for biological control and integrated pest management has been widely acknowledged, there is a striking lack of studies targeting the effects of learning by natural enemies on population and community dynamics and plant damage (for review, see Giunti et al., 2015; Little et al., 2019). Our study suggests that the release of learned natural enemies provides more efficient augmentative biological control than the release of target pest‐naïve natural enemies. The predator population founded by thrips‐naïve individuals could never catch up with the population founded by thrips‐experienced individuals, although one might have expected that any initial inter‐population difference arising from pre‐experimental individual learning should have gradually vanished due to progeny of the released individuals making similar experiences. The outcome of our experiment emphasizes that the success of biological control of an emerging pest population hinges on the quality of the released individuals, their immediate effects on the pests and their legacy regarding offspring quantity and possibly quality. Main population‐level factors responsible for better biological control were thrips‐experienced predators providing for a head start in population growth coupled with stronger suppression of the thrips population. These factors were most likely mediated by higher prey profitability and higher per capita predation rates, which, in turn, allowed a higher reproductive output, of the released thrips‐experienced predators than thrips‐naïve predators (Christiansen & Schausberger, 2017; Schausberger et al., 2018; Seiter & Schausberger, 2016). These effects were especially evident from week 1 to 2, when the predator population founded by thrips‐experienced individuals showed the steepest increase and the thrips population exposed to thrips‐experienced predators increased the least. Additionally, the offspring of the released thrips‐experienced predators may have been favoured by maternally induced prenatal priming on thrips prey relative to offspring of the released thrips‐naïve predators (Peralta‐Quesada & Schausberger, 2012). Peralta‐Quesada and Schausberger (2012) showed for *N. californicus* that mothers can transfer cues from their diet into the yolk of eggs, allowing prenatal learning to take place and shaping the diet preference of offspring. Assuming that thrips‐experienced predators were more inclined to attack and ingest thrips than thrips‐naïve predators, parental effects enhancing thrips recognition and acceptance by offspring were more likely to occur in thrips‐experienced than thrips‐naïve predators. Such transgenerational effects may have contributed to sustain differences in foraging behaviour between populations founded by thrips‐experienced and ‐naïve predators.

Due to size constraints and active defence abilities, mobile thrips stages are difficult to capture and overwhelm by predatory mites (e.g. Bakker & Sabelis, 1989; Reitz, 2009). This is especially true for small juvenile predatory mites. It is thus only the first, and occasionally second, larval stage of thrips that is attacked by predatory mites, mainly by gravid females. However, recent studies provided evidence that some predatory mite species including *A. swirskii* may circumvent the difficulties associated with capturing mobile thrips by going for thrips eggs, which are embedded in the leaf tissue (Nguyen et al., 2019). In our experiment, both thrips‐naïve and ‐experienced predatory mites had the strongest suppressing effect on thrips eggs followed by thrips larvae. Also, the difference in thrips suppression by thrips‐experienced and ‐naïve predators was most pronounced for thrips eggs, which provides a clue to the cognitive and behavioural changes brought about by thrips experience. Early learning by *A. swirskii* primarily shortens the time needed by the predators to recognize and attack thrips (Christiansen et al., 2016; Seiter & Schausberger, 2016), and thus represents a sort of perceptual learning (by imprinting and/or associative learning; Schausberger & Peneder, 2017; Seiter & Schausberger, 2016) rather than motor learning, that is, improved motor skills to capture and seize mobile thrips (Pearce, 2013). In our learning paradigm, the predators could not specifically learn how to better prey on thrips eggs because they had not been exposed to thrips eggs during the learning phase. Most likely, the predators learned a generalized thrips smell (cues on the thrips body and/or in metabolic waste products; Schausberger & Peneder, 2017; Schausberger, Seiter, et al., 2020) during the early life phase, when they were exposed to thrips larvae, which later allowed them to more easily find and better recognize thrips eggs embedded in the leaf tissue.

Our study provides indirect evidence that learned predatory mites are better able to counter the decrease in plant fecundity (which is an indicator of plant fitness from an evolutionary perspective and an important aspect of yield from the grower's perspective) caused by thrips herbivory. Plants produced more fruits (pods) with decreasing abundance of thrips and increasing abundance of predatory mites, whereby mite populations founded by learned individuals reached higher levels and suppressed thrips abundance more strongly than did populations founded by naïve individuals. Positive effects on yield may be an excellent argument to promote commercialization of learned natural enemies and the adoption of using learned natural enemies on a larger scale. Enhancing the biocontrol efficacy of natural enemies through targeted learning seems particularly promising for generalist predators. For example, mass‐rearing of many generalist predatory mites, including *A. swirskii* (Bolckmans & van Houten, 2006), takes place using factitious prey. Enriching such mass‐rearings with body extracts of thrips, which exposure may induce similar learning effects as does physical contact with whole thrips bodies (Schausberger, Seiter, et al., 2020), could allow production of thrips‐specific predator lines. *Amblyseius swirskii* is an omnivore that is currently used against various pest species but in most crops biological control of thrips is difficult and often insufficient (Mouden et al., 2017), calling for optimization efforts. Learned natural enemies may be costlier to produce, and may be more expensive in sale, than natural enemies reared in the standard way, due to the need of developing more sophisticated mass‐rearing techniques, yet may finally translate into economic benefits for biocontrol companies and growers applying those natural enemies.

## AUTHORS' CONTRIBUTIONS

P.S. conceived the study idea and designed the experiment; P.S., D.Ç. and A.L. conducted the experiment; P.S. analysed the data and wrote the manuscript. All authors approved submission of the manuscript.

## Data Availability

Data available via Dryad Digital Repository https://doi.org/10.5061/dryad.tr4v447 (Schausberger, Cekin, et al., 2020).
